# Quality assessment of systematic reviews of vitamin D, cognition and dementia

**DOI:** 10.1192/bjo.2018.32

**Published:** 2018-07-09

**Authors:** Fariba Aghajafari, Dimity Pond, Nigel Catzikiris, Ian Cameron

**Affiliations:** Assistant Professor, Department of Family Medicine, Cumming School of Medicine, University of Calgary Sunridge Family Medicine Teaching Centre, Canada; Professor and Head of Department of Family Medicine, University of Newcastle, Australia; Research Assistant, School of Medicine and Public Health, Faculty of Medicine, The University of Newcastle, Australia; Professor, Northern Clinical School, Rehabilitation Studies Unit, Sydney Medical School, The University of Sydney, Australia

**Keywords:** 25(OH)D, vitamin D, dementia, Alzheimer disease, cognition, systematic review, meta-analysis

## Abstract

**Background:**

There is conflicting evidence regarding the association of vitamin D with cognition performance and dementia.

**Aims:**

We aimed to summarise the evidence on the association of vitamin D with cognitive performance, dementia and Alzheimer disease through a qualitative assessment of available systematic reviews and meta-analyses.

**Method:**

We conducted an overview of the systematic reviews of all study types with or without meta-analyses on vitamin D and either Alzheimer disease, dementia or cognitive performance up to June 2017.

**Results:**

Eleven systematic reviews were identified, nine of which were meta-analyses with substantial heterogeneity, differing statistical methods, variable methodological quality and quality of data abstraction. A Measurement Tool to Assess Systematic Reviews checklist scores ranged from 4 to 10 out of 11, with seven reviews of ‘moderate’ and four of ‘high’ methodological quality. Out of six meta-analyses on the association between low serum concentration of 25-hydroxyvitamin D and risk of dementia, five showed a positive association. Results of meta-analyses on the association between low serum concentration of 25-hydroxyvitamin D and memory function tests showed conflicting results.

**Conclusions:**

This systematic evaluation of available systematic reviews provided a clearer understanding of the potential link between low serum vitamin D concentrations and dementia. This evaluation also showed that the quality of the available evidence is not optimal because of both the low methodological quality of the reviews and low quality of the original studies. Interpretation of these systematic reviews should therefore be made with care.

**Declaration of interest:**

None.

Dementia is a common problem worldwide, with 46.8 million people living with dementia in 2015.^1^ Although evidence from European countries indicates a reduction in age-specific incidence rate over time,^2^^–^^4^ prevalence is on the rise because of the aging population.^2^ Most people with dementia live in low- and middle-income countries and rates of increase are not uniform. It is estimated that the increase in prevalence of dementia in countries such as India, China and their south Asian and west Pacific neighbours will triple that of high-income countries by 2050.^1^^,^^2^ In addition, the risk of dementia is higher in women (22%) than men (14%) at age 65 years.^5^ Alzheimer disease is the most common type of dementia; it is estimated that the odds of receiving a diagnosis of Alzheimer disease after the age of 85 years exceed one in three.^6^ The cause of dementia and cognitive decline is complex, and effective treatment to prevent the progression of the disease is still lacking.^6^ Studies have shown the beneficial effect of dietary factors on cognitive function.^7^ Vitamin D is one of these dietary factors and has been suggested to beneficially affect cognition and memory.^7^ Vitamin D is a pro-hormone and is produced in the epidermis by 7-dehydrocholesterol as a reaction to sunlight.^8^ Nutrition is another important source of vitamin D.^8^ Therefore, preventive strategies targeting lifestyle and diet are essential to delay onset and slow decline.

Systematic reviews and meta-analyses of studies are regarded as the highest standard of scientific evidence and are a highly efficient way to pool data from several clinical studies and to inform the development of evidence-based clinical practice guidelines.^9^^,^^10^ Since 2009, at least ten systematic reviews of epidemiologic studies on the association between vitamin D and cognitive decline and dementia have been published.^7^^,^^11^^–^^20^ Although the results of these included meta-analyses suggest a positive association between lower vitamin D status and the development of dementia, there are differences in the quality of these systematic reviews, inclusion/exclusion criteria applied and the review/assessment methodology adopted.

The present overview was conducted to summarise the evidence on the association between vitamin D and cognitive performance, dementia and Alzheimer disease through a qualitative assessment of the available systematic reviews and meta-analyses.

## Method

To conduct this overview, we followed the Cochrane Collaboration methodology for overviews of reviews^21^ as well as the Preferred Reporting Items for Systematic Reviews and Meta-Analyses (PRISMA) reporting guidelines.^22^ In addition, we used the A Measurement Tool to Assess Systematic Reviews (AMSTAR) checklist to assess the quality of the systematic reviews.^23^^–^^25^

### Criteria for considering reviews for inclusion

We determined screening criteria *a priori*, with the aim of identifying fully published systematic reviews that assessed the association of vitamin D blood concentration or vitamin D supplementation and cognitive decline. We defined a systematic review as any review in which a comprehensive search strategy was specified *a priori* and an explicit and rigorous method was used to answer a research question. If the authors also quantitatively pooled results from the included studies, the systematic review included a meta-analysis. We excluded narrative reviews on the topics and systematic reviews that addressed wider questions of dietary/lifestyle factors on cognitive cognition with either previously published systematic reviews or one single study. If more than one original study was covered in the systematic reviews, then they were included in this overview.

### Search methods for identification of reviews

We performed a literature review in MEDLINE and Embase databases from inception to June 2017, in consultation with a senior research librarian. The search was broken into two themes:

- To identify vitamin D, a Boolean search was performed, using the term ‘or’ to explode (search by subject heading) and map (search by keyword) the following MeSH headings:
  ‘vitamin D’ OR ‘vitamin D2’ OR ‘vitamin D3’ OR ‘ergocalciferol’ OR ‘cholecalciferol’ OR ‘25-Hydroxyvitamin D’ OR ‘25(OH)D’ OR ‘25(OH)D2’ OR ‘25(OH)D3’ OR ‘3-epi-25 hydroxyvitamin D’ or ‘D2’ OR ‘D3’ OR ‘vitamin D Deficiency’ OR ‘hypovitaminosis D’.
- To identify dementia and cognitive decline, a second Boolean search was performed, using the term ‘or’ to explode (search by subject heading) and map (search by keyword) the following MeSH headings: ‘dementia’ OR ‘cognitive’ OR ‘cognitive disorders’ OR ‘Alzheimer’ OR ‘memory’ OR ‘memory, episodic’ OR ‘memory, long-term, OR ’memory, short-term’ OR ‘mental recall’ OR ‘recognition’ OR ‘repetition priming’ OR ‘retention’ OR ‘spatial memory’ OR ‘neuropsychological tests’ OR ‘executive function’ OR ‘psychomotor performance’ OR ‘global impairment’ OR ‘MMSE’ OR ‘mini mental state examination’ OR ‘attention’ OR ‘orientation’ OR ‘neuropsychological’ OR ‘brain’.

Themes (a) and (b) were combined with the Boolean operator ‘and’ to answer the focus questions. We used search strategies recommended for retrieving systematic reviews instead of limiting our search to ‘systematic review’ as a ‘publication type’, to optimise precision of the search.^26^

The search results were compiled with citation management software (Endnote version X7; Clarivate Analytics, http://clarivate.libguides.com/endnote).

### Data collection and analysis

Two independent reviewers (FA, NC) searched related articles and links. Additional articles were identified by manual search of the references from the key articles selected. Systematic reviews with or without meta-analyses on vitamin D and Alzheimer disease, dementia and cognitive and memory impairments were selected. Although the search was not limited by language, all systematic reviews included in the overview were published in English. Two independent reviewers independently assessed the abstracts for potential inclusion, fully reviewed the selected articles and selected the final articles for the overview. Studies that were only published as abstracts were excluded. Any disagreement was resolved by means of meeting and discussion among authors to establish a consensus.

### Data extraction and management

We developed a data extraction form to collect key indicators of each systematic review, including the number of articles in the systematic review, the number of articles in the meta-analysis, the number of participants in each review, inclusion criteria of the original articles, outcomes of interest, databases searched, design of the included articles, adjustment for potential confounders by the included articles, analytical approach, summary estimate, sensitivity and subgroup analysis and main conclusion of the systematic reviews. Two reviewers (FA, NC) independently extracted information from each article and compared findings; any discrepancies were resolved by consensus between the reviewers.

### Assessment of methodological quality of included reviews

We assessed the quality of the systematic reviews with the AMSTAR checklist.^23^ AMSTAR is a comprehensive, face-validated and content-validated tool to assess the methodologic quality of systematic reviews.^24^^,^^25^ We categorised AMSTAR scores as ‘high’ (8–11 out of a possible 11 points), ‘moderate’ (4–7 points) and ‘low’ (≤3 points) to classify the methodological quality of the identified reviews based on a previously published study.^27^ At any point, any disagreement between reviewers (FA, DP) was resolved by means of meeting and discussion among the authors to establish a consensus.

### Data synthesis

We extracted data from the included systematic reviews and prepared the data in table format. The review outcomes are described in the Results section.

## Results

### Description of included studies

**Fig. 1:**
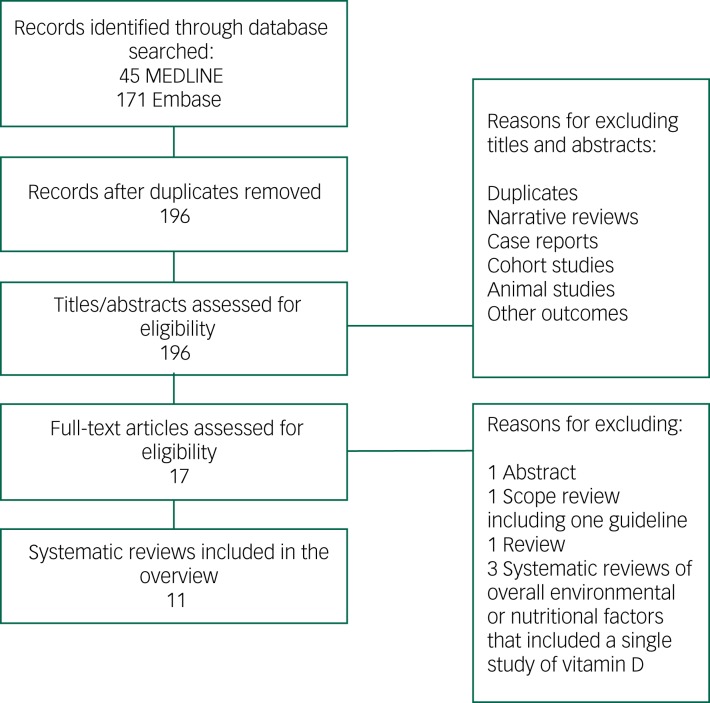
Flowchart of studies selected for overview of systematic reviews.

The results from the combined search totalled 196 citations after removal of duplicates. The title and abstract of these articles were screened, and 17 papers were selected for full review.^7^^,^^11^^–^^20^^,^^28^^–^^33^ After full-text screening of these articles, 11 were selected for the overview (Fig. 1).^7^^,^^11^^–^^20^ Articles were excluded for the following reasons: a general review,^28^ a scope review presenting a guideline,^29^ systematic reviews of overall environmental or nutritional factors affecting cognition and including a single study of vitamin D^30^^–^^32^ or published as an abstract.^33^ The number of studies included per systematic review ranged from 3 to 37.^7^^,^^11^^–^^20^ Publication dates of the included systematic reviews were between 2009^11^ and 2017.^20^ Although all 11 reviews included observational studies (cross-sectional, case–control and prospective), only three of these reviews included interventional studies.^12^^,^^15^^,^^16^ The interventional articles studied the effect of vitamin D (either alone or included within a supplement containing various other nutrients) on cognition. In nine reviews, the association between vitamin D and cognition was the primary question of interest.^11^^–^^18^^,^^20^ With two exceptions,^11^^,^^16^ the reviews performed a meta-analysis on a number of included studies (Table 1). Of the nine meta-analyses that reported on heterogeneity, five had a very high rate of heterogeneity among the included studies reported by *I*^2^ test (>50%).^12^^–^^15^^,^^17^

**Table 1:** Characteristics of the included systematic reviews

First author, year	Inclusion criteria	Outcomes	Assessment of dementia	Databases searched and search window	Articles included in review, articles included in meta-analysis	Number of patients	Design of the included articles	Main conclusion
Annweiler, 2009^11^	Data on serum 25(OH)D and cognitive status or diagnosed dementia, only studies with a healthy control group and use of a regression model for analysis in French and English	Cognitive status or diagnosed dementia	Not defined	MEDLINE, Cochrane library, PsychINFO between 1979 and December 2008	5 studies, N/A	19 597	Cross-sectional (*n* = 4), case–control (*n* = 1)	Inconclusive results on the association between serum 25(OH)D and cognitive performance
Annweiler, 2013^14^	Any type of observational/ interventional studies, data on serum 25(OH)D and Alzheimer disease, adult humans, written in Latin alphabet	Alzheimer disease	Not defined	MEDLINE, PsychINFO from inception to May 2012	10 studies, 7 studies	1005	Case–control (*n* = 7), nested case–control (*n* = 2), prospective (*n* = 1)	Patients with Alzheimer disease had lower serum 25(OH)D compared with matched controls
Annweiler, 2013^15^	Any type of observational/ interventional study, data on 25(OH)D and cognition, adult humans, written in Latin alphabet	Memory and executive dysfunction	N/A	MEDLINE, PsychINFO from inception to May 2012	17 studies, 12 studies	39 975	Cross-sectional (*n* = 11), prospective (*n* = 3), pre-post design (*n* = 2), RCT (*n* = 1)	Lower serum 25(OH)D concentration predicts executive dysfunction (mental shifting, information updating, processing speed), uncertain association with episodic memory
Balion, 2012^12^	Any type of observational/interventional studies, data on 25(OH)D, cognition and dementia, humans aged >18 years, written in English	Cognitive function, dementia	NINCDS–ADRDA, DSM-III or IV, MMSE	MEDLINE, Embase, AMED, Cochrane library, PsychINFO from inception to August 2010	37 studies, 4 studies (Alzheimer disease), 10 studies (MMSE test)	≅35 000	Cross-sectional (*n* = 21, case–control (*n* = 10), prospective (*n* = 2), pre-post design (*n* = 1), RCT (*n* = 3)	Lower 25(OH)D concentrations are associated with poorer cognitive function and a higher risk of Alzheimer disease
Cao, 2016^19^	Cohort studies including white patients, with follow-up for 1 year, data presented as risk or hazard ratios, with 95% CIs or with enough data to calculate these numbers, data on any type of dietary pattern or food consumption and dementia or mild cognitive impairment, on humans, and written in English	Dementia or mild cognitive impairment	Diagnostic codes	MEDLINE, Embase, BIOSIS, Cochrane library from 1997 to September 2014	3 studies, 3 studies	12 702	Prospective cohort (*n* = 3)	Low levels of vitamin D related to increase in dementia
Etgen, 2012^13^	Cross-sectional or longitudinal studies, at least 100 participants, data on vitamin D and cognitive impairment, results presented as odds or hazard ratios	Cognitive impairment	N/A	MEDLINE, Cochrane library, from 1980 to April 2012	15 studies, 7 studies	7688 in meta-analysis	Cross-sectional (*n* = 9), prospective (*n* = 2)	An increased risk of cognitive impairment in those with low vitamin D
Lopes da Silva, 2014^7^	Data on any type of plasma nutrient status and Alzheimer disease	Alzheimer disease	NINCDS–ADRDA, DSM III or IV	MEDLINE, Embase, Cochrane library, from 1990 to March 2012	5 studies, 5 studies	865	Case–control (*n* = 5)	No association between low levels of vitamin D and Alzheimer disease
Shen, 2015^18^	Data on 25(OH)D concentration with cut-off point of <50 nmol/L and Alzheimer disease or dementia, results presented as odds, risk, or hazard ratios, written in English	Alzheimer disease and dementia	Not defined	MEDLINE, from inception to February 2015	Alzheimer disease: 5 studies, 5 studies Dementia: 5 studies, 4 studies	Alzheimer disease: 10 019 Dementia: 5073	Alzheimer disease: cross-sectional (*n* = 1), prospective (*n* = 4) Dementia: cross-sectional (*n* = 3), prospective (*n* = 2)	Lower vitamin D status is associated with increased risk of developing Alzheimer disease and dementia
Sommer, 2017^20^	RCTs, prospective cohort and nested case–control, systematic reviews of longitudinal studies with data on the effect of sunlight exposure or vitamin D serum concentrations (as surrogate) and dementia, among adults, written in English and German	Dementia: Alzheimer disease vascular, frontotemporal, Lewy body	Based on validated measurement scales	MEDLINE, Embase, Cochrane library, SCOPUS, Web of Science, ICONDA, PsychINFO, the Open Grey database, from 1990 to October 2015	6 studies, 5 studies	18 933	Prospective (*n* = 5), retrospective (*n* = 1)	Vitamin D deficiency increases the risk of dementia
van der Schaft, 2013^16^	Observational studies with data on vitamin D (serum concentration or dietary intake) and cognition in adult humans, available measure of association	Cognition	Cognitive function test (e.g. MMSE, CDT)	Embase, PubMed from inception to June 2012	28 studies, N/A	59 576	Cross-sectional (*n* = 25), prospective (*n* = 6)	Hypovitaminosis D is associated with worse outcome on one or more cognitive function tests and a higher frequency of dementia
Zhao, 2013^17^	Comparative analysis of 25(OH)D of individuals with Alzheimer disease against healthy population	Alzheimer disease	Not defined	MEDLINE, from 1983 to March 2012	6 studies, 6 studies	892	Case–control (*n* = 6)	Patients with Alzheimer disease had non-significant lower levels of 25(OH)D relative to healthy controls

25(OH)D, 25-hydroxyvitamin D; AMED, Allied and Complimentary Medicine Database; BIOSIS, BioSciences Information Service of Biological Abstracts; CDT, Wolf–Klein Clock Drawing Test; ICONDA, International Construction Database; MMSE, Mini-Mental State Examination; N/A, not applicable; NINCDS–ADRDA, National Institute Neurological and Communicative Disorders and Stroke–Alzheimer Disease and Related Disorders Association; RCT, randomised controlled trial; SCOPUS, Elsevier abstract and citation database.

There was inconsistency of inclusion/exclusion criteria applied to studies in meta-analyses across the systematic reviews. For example, Shen and Ji^18^ did not include the previous cross-sectional studies that other reviewers^14^^,^^17^ had included in their earlier meta-analyses. There were also differences in the number of studies included in the meta-analyses across the three contemporaneously published studies.^12^^,^^14^^,^^17^ These findings could reflect differences in inclusion criteria, data sources searched and/or timing of the searches conducted within these reviews.

### Methodologic quality of the included reviews

Table 2 shows the AMSTAR quality scores of the included reviews. Of the 11 included systematic reviews, seven were assessed as having moderate methodological quality^7^^,^^11^^,^^13^^,^^16^^–^^19^ and four had high methodological quality.^12^^,^^14^^,^^15^^,^^20^ The key characteristics that distinguished studies with high AMSTAR scores included assessment of quality of the studies and assessment of publication bias. With one exception,^20^ all included studies declared conflicts of interest for the review but not for the included studies.

**Table 2:** A Measurement Tool to Assess Systematic Reviews (AMSTAR) checklist quality scores of the included reviews

AMSTAR questions	Annweiler, 2009^11^	Annweiler, 2013^14^	Annweiler, 2013^15^	Balion, 2012^12^	Cao, 2016^19^	Etgen, 2012^13^	Lopes da Silva, 2014^7^	Shen, 2015^18^	Sommer, 2017^20^	van der Schaft, 2013^16^	Zhao, 2013^17^
*A priori* design	Yes	Yes	Yes	Yes	Yes	Yes	Yes	Yes	Yes	Yes	Yes
Duplicate study selection and data extraction	Yes	Yes	Yes	Yes	Yes	No	No	Yes	Yes	No	Yes
Comprehensive literature review	Yes	Yes	Yes	Yes	Yes	No	No	No	Yes	Yes	No
Unpublished grey reports sought	No	No	No	No	No	No	No	No	Yes	No	No
List of included and excluded studies provided	No	Yes	No	No	No	No	No	No	No	No	No
Characteristics of individual studies provided	Yes	Yes	Yes	Yes	Yes	Yes	No	Yes	Yes	Yes	Yes
Scientific quality of studies assessed and documented	No	Yes, Newcastle–Ottawa Scale	Yes, Crombie criteria	Yes, Newcastle–Ottawa Scale, (Jadad)	No	No	No	No	Yes, Newcastle–Ottawa Scale	No	No
Scientific quality of studies used for conclusion	Yes	Yes	Yes	Yes	No	Yes	Yes	No	Yes	Yes	No
Statistical method appropriate	Yes	Yes	Yes	Yes	Yes	Yes	Yes	Yes	Yes	Yes	Yes
Likelihood of publication bias assessed	No	No	Yes	Yes	Yes	Yes	Yes	No	Yes	Yes	No
Conflict of interest declared in both systematic review and included studies	No	No	No	No	No	No	No	No	Yes	No	No
Aggregated scored	6/11	8/11	8/11	8/11	6/11	5/11	4/11	4/11	10/11	6/11	4/11

### Effect of interventions

#### Association between vitamin D status and Alzheimer disease and dementia

##### Cross-sectional and case–control studies

Of the seven meta-analyses conducted on the association between 25-hydroxyvitamin D (25(OH)D) concentration and risk of dementia or Alzheimer disease,^7^^,^^12^^,^^14^^,^^17^^–^^20^ five reviews showed an increased risk of dementia or Alzheimer disease with lower concentration of 25(OH)D (Tables 1 and 3).^12^^,^^14^^,^^18^^–^^20^ A comprehensive systematic analysis on the association between 25(OH)D concentration and risk of dementia and cognitive decline conducted by Balion *et al*^12^ included 37 studies. They included all studies with any recognised diagnostic criteria for dementia and accepted all validated neuro-psychological tests as a measure of cognitive function. The authors extracted data on study design, study setting, population characteristics, vitamin D type and assay method, cognitive measures and statistical methods. They conducted two meta-analyses, one comparing mean 25(OH)D between Alzheimer disease and a control group and the second comparing mean Mini-Mental State Examination (MMSE) scores between individuals with 25(OH)D <50 nmol/L and those with 25(OH)D ≥50 nmol/L. Six studies (case–control and cross-sectional) demonstrated a lower mean 25(OH)D concentration in patients with Alzheimer disease than in controls (−6.2 nmol/L; 95% CI −10.6 to −1.8), after adjusting for confounders. They concluded that differing vitamin D assay methods was the reason for heterogeneity among these studies.

**Table 3:** Assessment of meta-analytic approach and results of the included systematic reviews

First author, year	AMSTAR rating	Overall conclusion	Adjustment for potential confounder by the included articles (number of studies)	Analytic approach	Summary estimate (95% CI)	Sensitivity analysis	Subgroup analysis
Annweiler, 2009^11^	Moderate	Inconclusive association	No adjustment (*n* = 2), partial adjustment: age, gender, ethnicity, solar exposure education, serum vitamins B1, B6 and B12 (*n* = 3)	N/A	N/A	N/A	N/A
Annweiler, 2013^14^	High	Increased risk of Alzheimer disease	Partial adjustment in a few studies: age, gender, ethnicity, BMI, education, season, medical comorbidities, ApoE genotype, kidney function, plasma homocysteine, multivitamin use, home care centre	Fixed and random effects model with inverse variance method; effect size derived from mean, s.d. and size of each group	Summary effect size 1.40 (0.26–2.54), a large effect size (*P* = 0.016), *I*^2^ = 98%	N/A	N/A
Annweiler, 2013^15^	High	Decreased executive dysfunction, uncertain effect on episodic memory	Partial adjustment in ten studies: age, gender, BMI, ethnicity, education, income, health status, physical activity, alcohol, tobacco and caffeine consumption, season, centre, serum concentration of PTH, calcium, vitamin E and zinc	Fixed effect model; effect size derived from mean, s.d. and size of each group	1. Episodic memory; effect size −0.09 (−0.16 to −0.03), *I*^2^ = 86% 2. Executive dysfunction (mean difference of scores); processing speed: 4.01 (1.20–6.83), *I*^2^ = 96%; mental shifting: 12.47 (6.78–18.16), *I*^2^ = 91%; information updating tests −0.45 (−0.60 to −0.29), *I*^2^ = 69%	N/A	N/A
Balion, 2012^12^	High	Increased risk of Alzheimer disease, decreased cognitive function	Partial adjustment in 12 studies for season, sunlight exposure, site/centre, alcohol, smoking, BMI, baseline cognitive score, comorbidities, physical activities, physical performance, education, energy intake, multivitamin use, vitamin E, race, ethnicity, depression, psychoactive drugs, kidney function, biomedical measure of albumin, ApoE, vitamins B1, B6 and B12, calcium, homocysteine and PTH	Random effects model: using weighted mean difference and Hedge's *g*-test	1. Alzheimer disease (*n* = 888): weighted mean difference: −6.2 (−10.6 to −1.8), *I*^2^ = 0% 2. MMSE (*n* = 2749) effect size 1.2 (0.5–1.9) *I*^2^ = 65%	Two studies were excluded by CPBA assay, change in heterogeneity and overall estimate	*A priori* subgroup analysis: no change in MMSE point estimate
Cao, 2016^19^	Moderate	Increased risk of cognitive decline	Adjustment on three studies in the meta-analysis (age, gender, race, season, BMI, physical activity, education, income, hypertension, depression, smoking and alcohol consumption)	Random effects model	Risk ratio 1.52 (1.17–1.98) *I*^2^ = 45%	N/A	N/A
Etgen, 2012^13^	Moderate	Increased risk of cognitive impairment	Partial adjustment of all the seven studies for age, gender, race, season, BMI, physical activity, education, income, comorbidities, measure of albumin, ApoE, vitamins B1, B6 and B12, calcium, homocysteine, PTH, zinc, smoking, alcohol consumption and pulse pressure	Random effects model	Odds ratio 2.39 (1.91–3.00) *I*^2^ = 56%		Grouped studies based on study characteristics: gender, cognitive function test, sample size <500, community dwelling, in-patient, no change in overall estimate
Lopes da Silva, 2014^7^	Moderate	No association between low levels of vitamin D and Alzheimer disease	Adjustment for age in meta-analysis	Random effects model fitted by restricted maximum likelihood	Weighted mean difference −5.77 (−12.11 to 0.58), *P* = 0.075	Adjusted based on age, change in point estimate from significant to non-significant	N/A
Shen, 2015^18^	Moderate	Increased risk of Alzheimer disease and dementia	Partial adjustment of all ten studies for age, gender, season, sunlight exposure, alcohol, smoking, BMI, comorbidities, physical activities, physical performance, education, race, ethnicity, depression, ApoE, income, creatinine, cholesterol and plasma homocysteine	Random effects model	Alzheimer disease: odds ratio 1.21 (1.02–1.41) *I*^2^ = 0%; Dementia: odds ratio 1.49 (1.09–1.88) *I*^2^ = 0%	N/A	N/A
Sommer, 2017^20^	High	Increased risk of dementia	Adjustment of all six studies	Fixed and random effects model with generic inverse variance model	Point estimate 1.54 (1.19–1.99) *I*^2^ = 20%	Examination of heterogeneity based on different confounding factors, no change in estimates	N/A
van der Schaft, 2013^16^	Moderate	Increased risk of dementia and worsening cognitive function	No adjustment (*n* = 6), partial adjustment for age, education, BMI and gender (*n* = 22)	*χ*^2^ exact test to analyse association between study variables and outcome direction	71% of studies showed positive association between vitamin D level and cognitive function	N/A	N/A
Zhao, 2013^17^	Moderate	No association between low levels of vitamin D and Alzheimer disease	No adjustment	Random effects model	Summary mean difference −1.39 (−2.79 to 0.01), *I*^2^ = 98%	N/A	N/A

AMSTAR, A Measurement Tool to Assess Systematic Reviews; ApoE, Apolipoprotein E gene; BMI, body mass index; CPBA, competitive protein binding assay; MMSE, Mini-Mental State Examination; N/A, not applicable; PTH, parathyroid hormone.

Another comprehensive systematic review and meta-analysis found similar associations. In their review, Annweiler *et al*^14^ included ten observational studies with an outcome of diagnosis of Alzheimer disease, regardless of the severity, duration or management of the disease. The authors performed a meta-analysis on seven case–control studies totalling 357 cases and 648 controls. The results were expressed as a bias-corrected ‘effect size’ of the difference between serum 25(OH)D concentrations in cases and controls and derived from the mean, s.d. and size of each group. Any size greater than 0.8 was described as large. The summary random effect size from meta-analysis was 1.40 (95% CI 0.26–2.54), indicating that serum 25(OH)D concentrations were overall 1.4 s.d. lower in Alzheimer disease cases compared with controls (*P* = 0.016), which represents a strong association of low 25(OH)D concentration with Alzheimer disease. However, the meta-analysis had very high heterogeneity (*I*^2^ = 98%).

A non-significant association was evident in another meta-analysis on hypovitaminosis D and Alzheimer disease. Zhao *et al*^17^ performed a meta-analysis on six studies encompassing 319 patients and 573 controls, showing that patients with Alzheimer disease had lower levels of 25(OH)D (summary mean difference (SMD) −1.39; 95% CI −2.79 to 0.01), with a very high heterogeneity (*I*^2^ = 98%). Similarly, a meta-analysis of five observational studies by Lopez *et al*^7^ found that patients with Alzheimer disease had non-significant lower concentrations of 25(OH)D compared with controls after adjustment for age (SMD −5.66; 95% CI −12.11 to 0.58).

##### Longitudinal studies

Shen and Ji^18^ performed a meta-analysis on a number of recent prospective cohort studies that were not available in previous reviews and demonstrated a significant increase in risk of Alzheimer disease in patients with 25(OH)D ≤50 nmol/L (odds ratio 1.21; 95% CI 1.01–1.40) and a significant increase in risk of developing dementia (odds ratio 1.63; 95% CI 1.09–2.19), with no significant heterogeneity.

The most recent meta-analysis conducted by Sommer *et al*^20^ focused on longitudinal studies, including five prospective cohort studies and one retrospective cohort study with follow-up ranging between 2 to 21 years. Although the meta-analysis intended to evaluate the effect of sunlight exposure on dementia risk, vitamin D concentration was used as a surrogate parameter for sunlight because of the lack of any studies on the subject. They performed meta-analysis on five studies totalling 18 933 patients with all types of dementia, and showed that persons with vitamin D concentration <25 nmol/L are at higher risk of dementia compared with persons with vitamin D concentration ≥50 nmol/L (point estimate 1.54; 95% CI 1.19–1.99), with no significant heterogeneity (*I*^2^ = 20%). Sensitivity analysis based on different confounding factors, including vitamin D assay method and cut-point level, did not show any change in the total estimates. The authors also used the Grading of Recommendations, Assessment, Development and Evaluation (GRADE) system^34^ for assessing the quality of the evidence in the conclusion. They rated the evidence very low because of the observational nature of the included studies, lack of control for important confounding factors across studies and use of serum vitamin D concentration as surrogate for sunlight exposure.

Table 4 shows the strength of the evidence on the effect of vitamin D deficiency on risk of Alzheimer disease, using the GRADE system.^34^ The strength of the evidence is very low mainly because of the observational nature of the studies, which did not consider all of the important confounding factors.

**Table 4:** Grading of Recommendations, Assessment, Development and Evaluation (GRADE) evidence profile of the included systematic reviews

Participants (studies)	Risk of bias	Inconsistency	Indirectness	Imprecision	Publication bias	Overall quality of evidence
Seven systematic reviews on Alzheimer disease	Very serious	Not serious	Not serious	Serious	All plausible residual confounding would reduce the demonstrated effect	⨁◯◯◯ Very low
Six systematic reviews on cognitive function	Very serious	Not serious	Not serious	Serious	All plausible residual confounding would reduce the demonstrated effect	⨁◯◯◯ Very low

#### Association between vitamin D status and cognitive function

##### Cross-sectional and case–control studies

Epidemiologic studies have shown the possible involvement of vitamin D in physiological brain processes. Annweiler *et al*^11^ conducted a comprehensive review of the literature and provided evidence on the association between low vitamin D status and global cognitive impairment. They looked at all cognitive tests, such as the MMSE, Clinical Dementia Rating Scale and Abbreviated Mental Test. In a more recent paper,^15^ they updated the review and performed a meta-analysis of the studies on episodic memory and executive function in adults. They extracted data on study design, setting and study populations, vitamin D and cognitive assessment methods and description of vitamin D and domain-specific cognitive outcomes. The authors pooled all studies addressing the same domain-specific function in people with higher or lower serum 25(OH)D concentrations. The definition of higher or lower 25(OH)D concentration varied depending on the study and population, and was based on threshold values defined *a priori* or on population-based quantiles. Studies included were predominantly observational in design (case–control, cross-sectional and prospective longitudinal) but included several interventional studies. The included studies covered a wide range of age, gender and race. Some studies adjusted for confounding factors like age, gender, body mass index (BMI), ethnicity, education, income, health status, physical activity, mobility, disability, renal function, alcohol, tobacco and caffeine consumption, centre and season. The tests evaluating episodic memory explored both verbal and visual memory and the tests evaluating executive function either provided a global approach or explored information processing through different tests.

Episodic memory refers to the processes of encoding, storage and retrieval of information personally experienced. Annweiler *et al*^15^ performed meta-analysis of cross-sectional studies and demonstrated a summary effect size of −0.09 (95% CI −0.16 to −0.03), suggesting that the scores on a variety of tests exploring episodic memory among 2105 participants were 0.09 s.d. lower in those with lower serum 25(OH)D concentrations compared with those with higher serum 25(OH)D concentrations. However, the authors stated that this summary effect size (−0.09) represented a small association and did not reach the threshold magnitude of 0.25 deemed to be an ‘educationally significant’ effect, as defined by Wolf^35^ (e.g., something is learned).

Executive function refers to a heterogeneous set of high-level processes that control and regulate other abilities and behaviours, and each of its subdomains can be tested separately with specific psychometric measures. In regard to processing speed, Annweiler *et al*^15^ conducted a meta-analysis of three studies encompassing 350 participants and showed a better Trail Making Test, Part A performance in participants with higher serum 25(OH)D concentrations (SMD 4.04; 95% CI 1.20–6.83). In addition, Digit Symbol Test scores improved linearly with the increase in 25(OH)D concentration (summary regression coefficient *β* = 0.03 for 1 ng/mL; 95% CI 0.01–0.05). In regard to mental shifting, meta-analysis of four studies with 802 participants demonstrated a better performance on Trail Making Test, Part B score in participants with higher serum 25(OH)D concentrations (SMD 12.47; 95% CI 6.78–18.16).

Another comprehensive review by Balion *et al*^12^ included data from eight cross-sectional and case–control studies with a total of 2749 participants, comparing MMSE scores between participants with 25(OH)D concentration <50 nmol/L and those with 25(OH)D ≥50 nmol/L. This review demonstrated a higher average MMSE score with higher vitamin D concentration (average difference 1.2; 95% CI 0.5–1.9) with a significant heterogeneity (*I*^2^ = 0.65).

Etgen *et al*^13^ included five cross-sectional studies with 5686 participants in a meta-analysis that showed a significant increased risk of cognitive impairment in those with vitamin D deficiency (odds ratio 2.37; 95% CI 1.77–3.17). Cognitive impairment was measured by different tests across the studies, and vitamin D deficiency was defined as 25(OH)D <25 nmol/L in three studies and <50 nmol/L in two studies. There was a significant heterogeneity among the included studies (*I*^2^ = 0.67) mainly because of the differences in participants' age and physical activity.

A systematic review by van der Schaft *et al*^16^ showed a significantly worse outcome on one or more cognitive function tests or a higher risk of dementia with lower vitamin D concentrations or intake in 18 out of 25 (72%) cross-sectional studies.

A recent systematic review and meta-analysis of dietary pattern and risk of dementia^19^ included three studies on the association between vitamin D on dementia and demonstrated that a lower level of vitamin D was associated with cognitive decline as measured by different psychological tests (risk ratio 1.52; 95% CI 1.17–1.98).

##### Longitudinal studies

Meta-analysis of longitudinal studies by Annweiler *et al*^15^ that included 4095 participants with a mean follow-up of 4.4 years found a decline in Trail Making Test, Part B scores for participants with lower 25(OH)D concentrations at baseline. They also showed the scores on a variety of tests exploring information updating were lower in participants with lower serum 25(OH)D concentration (summary effect size −0.31; 95% CI −0.53 to −0.09). Furthermore, they performed meta-analysis of interventional studies on the effect of vitamin D supplementation on executive function among 234 participants and showed a modest pre-post effect of vitamin D supplement on executive function scores in participants taking supplements (summary effect size −0.50; 95% CI −0.69 to −0.32). However, there was not a significant effect size at the end of the follow-up period in both the supplementation and control groups (summary effect size 0.14; 95% CI −0.04 to 0.32).^15^

In the meta-analysis conducted by Balion *et al*,^12^ which included two cohort studies, there were conflicting results between vitamin D status and MMSE scores; there were also two randomised studies with no significant effect of vitamin D supplementation on MMSE score.

When Etgen *et al*^13^ pooled the results from two longitudinal studies, vitamin D deficiency was significantly associated with increased risk of incident cognitive impairment (odds ratio 2.49; 95% CI 1.74–3.56) with no heterogeneity (*I*^2^ = 0.1). They also included two interventional studies in their review. In a randomised controlled trial, one dose of intramuscular injection of 600 000 IU ergocalciferol improved choice reaction time compared with placebo in elderly people with a history of falls. However, in a prospective study, multiple oral doses of 50 000 IU ergocalciferol for 4 weeks did not improve neurocognitive performance in nursing home residents.

In addition, a systematic review by van der Schaft *et al*^16^ showed that in four out of six (66.7%) prospective studies, there was higher risk of cognitive decline after a follow-up period of 4–7 years in participants with lower vitamin D concentrations at baseline.

Table 4 shows the strength of the evidence on the effect of vitamin D deficiency on cognitive dysfunction, using the GRADE system.^34^ The strength of the evidence is very low, mainly because of the observational nature of the studies, which did not consider all of the important confounding factors.

## Discussion

### Summary of main results

Available data, including evidence synthesis, provide evidence on the potential association between vitamin D status and Alzheimer disease, dementia and cognitive impairment. We systematically assessed evidence of the association between serum 25(OH)D concentration and the risk of Alzheimer disease, dementia and cognitive impairment among published systematic reviews of observational studies. Variations in study characteristics, analytical methods and methodological qualities were identified, as well as differences in observational studies included in each review. Although the majority of the systematic reviews included cross-sectional studies, which have the inherent problem of reverse causality, a few systematic reviews that included longitudinal studies still showed a positive association between low vitamin D status and dementia and cognitive impairment. Overall, the causality from these findings cannot be inferred because of the observational nature of the included studies in the systematic reviews.

### Overall completeness

We also noticed some variability in the inclusion of observational studies across different reviews. Although more recent reviews included additional studies that would not have been available to authors of earlier reviews, this did not explain all of the variability observed. Factors such as different definitions of outcomes, differences in the performance of literature searches and contact with original authors to obtain more information may also have contributed to differences in the inclusion of studies.

### Quality of evidence

A systematic review attempts to collect all evidence that fits predefined eligibility criteria to answer a specific research question. Meta-analyses are conducted to assess the strength of the evidence and provide a single summary estimate of the effect. Based on Meta-analysis of Observational Studies in Epidemiology criteria,^36^ a systematic review needs to include a clearly stated set of objectives that include the study population, the condition of interest, the exposure or intervention and the outcome. It needs to describe a systematic search that aims to identify all studies that meet the eligibility criteria as well as an assessment of the validity of findings of the included studies (risk of bias), an assessment of heterogeneity and a clear description of statistical methods.^10^ We used AMSTAR rating scores to assess the quality of the systematic reviews included in this study. Overall, the reviews included within the overview had moderate to high methodologic quality. Although the majority of these reviews link vitamin D status to dementia and cognitive impairment, they mostly reported a high percentage of heterogeneity among the included studies. Only four out of nine reviews with meta-analysis^7^^,^^12^^,^^13^^,^^20^ made an effort to further explore the origin of the heterogeneity among the included studies.

Seven out of 11 reviews did not assess the quality of the included studies beyond the applied inclusion criteria.^7^^,^^11^^,^^13^^,^^16^^–^^19^ Almost all included studies were observational, with the majority involving a cross-sectional or case–control design. Overall estimates from observational studies may be more variable than estimates from randomised trials of a similar sample size, reflecting the noise introduced by uncontrolled aspects of the study design.^37^ Furthermore, causality between vitamin D status and dementia cannot be determined through cross-sectional studies. As noted in some of these reviews,^12^^,^^14^^,^^20^ it is impossible to rule out reverse causality as an alternative explanation, i.e. low 25(OH)D concentration may be a result of the physiologic disturbances associated with the disease and/or its treatment, such as reduced exposure to sunlight and modification in dietary habits due to hospital admissions, treatments, reduced mobility and impairment in activities of daily living, including food preparation and other changes in lifestyle, all of which contribute to lowering 25(OH)D. A more recent systematic review^20^ attempted to address this issue by limiting their review to longitudinal studies, with a follow-up range between 2 and 21 years. These studies used a single measurement of serum 25(OH)D at baseline and followed participants over time to determine if they developed any cognitive impairment. However, the use of a single measurement of serum vitamin D concentration cannot be representative of long-term vitamin D status in participants,^37^ nor was it established whether these blood samples were taken at the critical time period during which vitamin D is more likely to affect cognition. Previous studies have raised the possibility of critical periods in which human cognition may be affected by vitamin D.^38^

Overall, these systematic reviews were limited by lack of or inconsistency in reporting confounding factors. A few systematic reviews reported information on the assay used to measure vitamin D concentrations.^11^^,^^12^^,^^14^^,^^15^^,^^20^ One review found that the method of 25(OH)D measurement was an important determinant of heterogeneity.^12^ This has been reported in other systematic reviews of vitamin D and disease-related outcomes.^39^ The method of vitamin D measurement is an important factor, as DEQAS (the International Vitamin D External Quality Assessment Scheme) has reported a range of inter-method variability for identical blood samples.^40^^,^^41^ Currently, the best assay to measure different types of vitamin D including epimers is liquid chromatography–tandem mass spectrometry.^42^ Studies in paediatric and adult populations have shown that not separating epimers from vitamin D estimation could overestimate vitamin D status.^42^^,^^43^ In addition to the inherent flaws in the assay methodology, the studies varied in their definition of cut-offs for 25(OH)D deficiency (>25 nmol/L and >50 nmol/L). Moreover, there are other confounding factors that may affect vitamin D status, including exposure to ultraviolet B light, latitude, season, ethnicity, nutritional status, BMI and vitamin D receptor genotype.^8^ Furthermore, there are multiple factors affecting dementia/cognitive impairment, such as age, gender, other comorbidities, education and living situations.^44^ These parameters were absent or partially evaluated in most studies included in the systematic reviews evaluated. Finally, in the assessment of memory, discrepancies exist among the original studies on the type of cognitive measure assessed.

### Strengths and limitations

One strength of this overview is the use of AMSTAR to assess methodological quality of the included systematic reviews with or without meta-analysis. Future systematic reviews could follow the established reporting guidelines.^22^ In particular, assessment of quality of included studies, assessment of publication bias and the presentation of conflict of interest were infrequently present in the studies included in this review.

Our overview is limited by those of the included studies. We encountered difficulty in combining the point estimates of the meta-analysis. We chose to present the range of point estimates across included reviews to obtain overall qualitative appraisal of the evidence, as well as to provide an opportunity to show the wide range and heterogeneity of the evidence.

Since the publication of the most recent systematic review^20^ included within this overview, a number of relevant studies have been published, with most being in agreement with our overall findings. However, they have significant limitations, including weak observational methods (case–control) and limited adjustment of confounding factors.^45^^,^^46^ The results from interventional studies are also conflicting, and these studies are limited by dose/type of vitamin D supplement and duration of follow-up.^47^^–^^49^

Based on the systematic evaluation of the previous literature in the field, there may be an association between vitamin D status and dementia and cognitive impairment. However, little is known about the function of vitamin D in relation to different cognitive domains. This systematic evaluation of previous systematic reviews provides a clear view of the quality of evidence on the association between low concentration of vitamin D and dementia. We also showed that the quality of the available evidence is not always optimal because of lower methodologic quality of the reviews and the low quality of the original studies. Inconsistencies in cognitive assessment methods, study populations, definition and measurement of vitamin D deficiency and reporting of confounders, as well as an undetermined potential for reverse causality, were the most prominent methodologic limitations in the studies included in this overview. Therefore, the strength of the evidence is low and interpretation of the systematic analyses discussed in this review should be made with caution. However, these reviews and the present overview provide analysis of the methodological issues that future supplementary studies on the topic need to consider in their research design.
